# Laser Drilling in Alumina Ceramics Using a Combination of Laser Pulses in the Free-Running and Q-Switched Modes

**DOI:** 10.3390/ma16093457

**Published:** 2023-04-28

**Authors:** Gennady Gavrilov, Andrey Kurkin, Evgeny Rusin, Evgeny Bazhenov

**Affiliations:** 1 Department of Materials Science, Materials Technology and Heat Treatment of Metals, Nizhny Novgorod State Technical University n.a. R.E. Alekseev, 603155 Nizhny Novgorod, Russia; 2 Department of Applied Mathematics, Nizhny Novgorod State Technical University n.a. R.E. Alekseev, 603155 Nizhny Novgorod, Russia; 3 Institute of Mechanical Engineering Problems, Russian Academy of Sciences, 603024 Nizhny Novgorod, Russia

**Keywords:** ceramic materials, laser drilling, millisecond laser, free-running mode, nanosecond laser, Q-switched mode, combined pulsed laser action, radiation pulse energy

## Abstract

The possibility of using a two-pulse laser action consisting of a pulse of the free-running pulse followed by the Q-switched pulse in the processes of laser drilling in alumina ceramics is considered. A diagram of a laser machine for implementing the proposed processing method and the method for determining the energy required to form the through hole and the drilling time are presented. For thermal exposure, pulsed radiation of Nd:YAG of the laser operating in the free-running mode (pulse duration 0.5 ms, energy up to 0.3 J) was used. The Q-switched pulse was generated using a second Nd:YAG laser (pulse duration 25–30 ns, energy up to 0.03 J). The laser radiation was focused on the surface of the material at one point. The time between the start of generation of the first and second lasers varied in the range of 0–1000 μs. The optimal delay time for the start of the generation of the second laser relative to the first was determined experimentally. The obtained results showed that the combination of the free-running pulse and the Q-switched pulse can significantly increase the efficiency of laser machining of ceramics and improve the hole quality. This scheme turned out to be promising for optimizing parameters of the process of laser drilling in thin-sheet alumina ceramics. The obtained results obtained have great potential in the field of precision laser machining of ceramics.

## 1. Introduction

The defining feature of the present time has been the transition to fundamentally new material processing technologies in many areas of production. The emergence of such technologies is due to not only the requirement to obtain new types of products with previously unknown complexes of properties, but also the need to save materials and energy. Modern production needs products with increased performance characteristics. Their production using traditional methods often causes significant technological difficulties. Since the possibilities of classical processing technologies are largely exhausted, the improvement, creation and development of new technologies are of particular relevance, a special place among which is occupied by laser processing [1,2,3]. 

Laser processing of materials is a well-established technology and has been used for more than four decades, playing an important role in the modern manufacturing industry and economy [4]. The technologies based on the laser processing methods, such as hardening [5], machining (cutting and drilling) [6], welding [7], marking [8], laser cladding [9] and laser alloying [10], have achieved significant success and are used in industry for the manufacture of machine parts and mechanisms made of materials of various physical nature. Laser processing has found wide application in many leading fields of industry, such as microelectronics, electrical engineering, aviation and mechanical engineering. In recent years, laser processing has played an increasingly significant role in improving product quality, labor productivity and production automation, as well as in reducing material consumption and environmental pollution [11,12]. The recent advances in laser technology are reflected in the research [2,13,14].

The important place among laser technologies is occupied by laser machining, which includes laser cutting and drilling. Compared with traditional mechanical drilling, electromechanical and ultrasonic drilling [15,16], laser drilling can be used for processing products with different geometries with a high degree of automation. Focused laser radiation, providing a high concentration of energy in the affected area, allows cutting operations and drilling in metal and ceramic materials, as well as in glasses, plastics and composites [17,18,19]. These processing technologies have a number of unique advantages associated with the possibility of implementing high-precision processing of materials with high surface quality in local impact zones [20,21]. The minimum heat-affected zone, the absence of mechanical action on the processed material, as well as the absence of temporary (during drilling) and residual stresses (after complete solidification) reduce the likelihood of microcracks and the other defects on the inner surface of the formed holes. High manufacturability and flexibility for controlling radiation parameters allow the performance of laser drilling along a complex contour of blanks of various shapes with a high degree of automation of the process. It should be noted that, for technologies of laser machining of metal and ceramic materials, repetitively pulsed solid-state laser installations are of the greatest interest [22]. This is due to the high quality of radiation, high performance, small dimensions, ease of maintenance and reliability [23]. By changing the laser parameters, such as wavelength, pulse energy, repetition rate, pulse duration and shape, and the area of the focus spot, it is possible to control the laser exposure process and ensure high quality of the holes. The effect of laser radiation on materials during drilling is characterized by general provisions related to the absorption and reflection of radiation and the spread of absorbed energy through the volume of the material due to thermal conductivity. The drilling of holes in materials is carried out via laser pulses operating in the free-running mode with the following main radiation parameters: the power flux density in the range of 10^6^–10^8^ W/cm^2^, and the duration of the radiation pulse in the range from 10^−4^ s to 10^−3^ s. The process of hole formation consists of two important stages. At the first stage, the surface is heated to the melting point and the melt bath is formed. Further absorption of radiation leads to the movement of the melting phase boundary deep into the material. At the second stage, when the surface of the melt reaches the temperature of intense evaporation, the energetic displacement or active release of the melt occurs due to the pressure of the vapors formed. The main parameters determining the performance of the hole formation process are the volume of the molten metal (or the penetration depth of the melting isotherm) and the amplitude of the vapor pressure pulse. Laser cutting and drilling methods, including in ceramics, are based on this principle of interaction of radiation with matter [24,25]. 

In a previous work [26], two nanosecond double-pulse lasers with the wavelengths of 355 and 532 nm, respectively, were used to form a combined dual-wavelength double-pulse train for the hole drilling and obtained a series of holes on stainless steel sheets. By using such a dual-wavelength double-pulse train, the area ratio of the front surface to the back surface is smaller than those using both a single-wavelength double-pulse train and a single-pulse laser, and the surface state of drilled holes has not changed significantly. The results show that the dual-wavelength double-pulse approach is the most effective method of the hole drilling with a small front-to-back area ratio among the three cases.

In another study [27], the new combined laser pulse (CLP) method was proposed for drilling alumina ceramic consisting of a millisecond (ms) pulse and the auxiliary nanosecond (ns) pulse sequence. 

In a study [28], theoretical and experimental results of drilling the alumina ceramic with thicknesses of 5 mm and 10.5 mm using millisecond (from 1 ms to 6 ms) pulsed Nd:YAG laser were reported.

In another research [20], the percussion drilling of alumina ceramics was performed using Nd:YAG laser and quasi-continuous-wave fiber laser. The effects of laser wavelength, pulse energy, pulse length and number of pulses were examined, and the comparison of produced holes geometry was reported. 

The combination of excellent physical and mechanical properties, such as high temperature resistance, high hardness and wear resistance, low thermal conductivity and electrical conductivity and chemical stability, has stimulated the use of ceramic materials in many modern industries. However, the high hardness and fragility of ceramics significantly complicates its processing with traditional methods, which is associated with significant energy consumption and high labor intensity, and the damage caused to the surface during processing affects the efficiency and service life of manufactured components. In addition, the processing of ceramics is accompanied by extreme tool wear associated with high processing loads, high vibration levels and long processing times. Therefore, machining processes often become impractical for manufacturing technologies of technical ceramic products. The contactless methods such as laser processing, ultrasonic processing, plasma processing, electron beam processing, electrochemical processing, etc. have become promising for processing ceramics [29,30,31]. With the development and improvement in industrial laser technologies, the laser processing method has become one of the most important for drilling micro-holes in structural ceramics. Laser machining of ceramics is currently one of the priority methods for the production of machine elements and mechanisms for the electronic and aerospace industries. 

The traditional methods of laser machining do not allow obtaining a high-quality calibrated hole in both metal and ceramic materials without internal surges and burr on the inner surface of the formed hole. The taper in most cases is k = 0.6–0.8, which is caused by the uneven distribution of laser radiation in the caustic of the focusing lens. The main measures aimed at eliminating these shortcomings and improving the technological parameters of laser drilling are considered [32,33,34]. In this work, to solve the previously mentioned problems, a laser processing scheme was proposed in which the free-running laser pulse performs the function of the heat source and ensures the symmetry of the melt zone, and in the final phase of exposure to the target creates conditions for the effective release of the condensed phase via an intense pressure pulse. This stimulates the removal of the burr from the hole and creates conditions for giving it a geometric shape close to cylindrical. Such conditions can be satisfied using the complex laser pulse with an inhomogeneous amplitude–time structure of radiation, which can be obtained from two laser generation modes—free-running and Q-switched modes. The implementation of this solution can be carried out by summing the pulses of free-running and Q-switched modes by reducing them to one point. In addition, it is possible to implement a method in which the Q-factor of the laser is switched during the action of the laser radiation pulse, consistent with the process of hole formation [35]. The novelty of the work was to determine the optimal time interval between the action of the pulse in the free-running mode and the subsequent pulse of the Q-switched mode, leading to more efficient removal of the formed hole of the influx and improving the quality of the morphology of the lateral surface. In addition, the objective of the study was to experimentally determine the time of hole formation and the minimum energy of the free-running pulse, which can serve as a useful addition to theoretical models of laser machining of ceramic materials.

## 2. Materials and Methods

**Figure 1:**
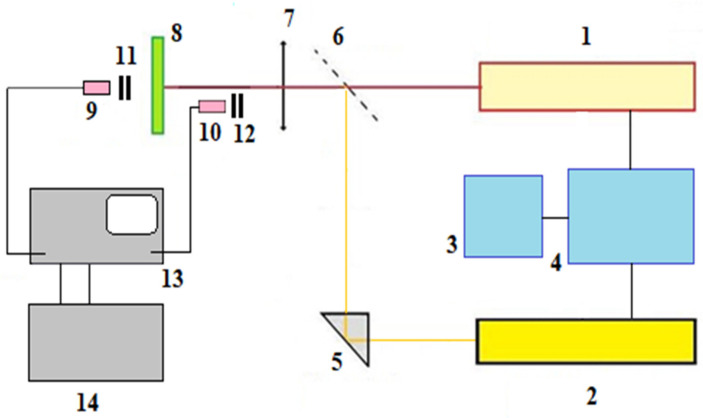
Diagram of equipment for combined laser machining of thin-sheet ceramics. 1—Nd:YAG laser in the free-running mode, 2—Nd:YAG laser in the Q-switched mode, 3—master oscillator, 4—two–channel pulse generator G5-27A, 5—prism, 6—partially transmitting mirror, 7—focusing lens, 8—sample, 9,10—photosensors, 11,12—selective filters (λ = 1.06 microns), 13—oscilloscope TDS 2004B, 14—personal computer.

For the experiments, the equipment was used, the main elements of which were Nd:YAG lasers with combined optical axes, one of which operated in the free-running and Q-switched modes (Figure 1). The aspherical lens with a focal length of 127 mm and the diameter of 38.1 mm was used to focus the laser radiation. Such lenses are usually used in cases where the smallest size of the focus spot is required, for example, when drilling ceramics. Aspherical lenses provide high values of specific power on the sample compared to the other types of lenses (for example, with Plano-convex) with equivalent focal lengths. The lens was mounted on the standard mount with the possibility of precision adjustment along three axes (X, Y, and Z). Focusing was carried out by moving the focusing lens.

**Figure 2:**
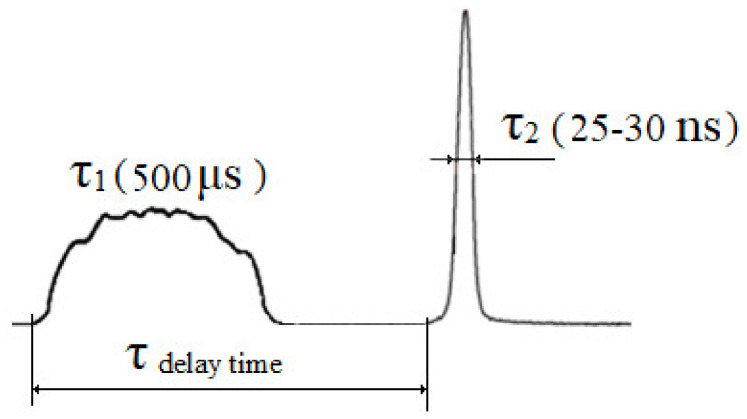
Qualitative image of a pulse with an inhomogeneous amplitude–time structure.

The important element of the equipment is the control unit, which made it possible to synchronize the operation of the lasers by changing the delay time of the trigger pulse of one laser relative to the beginning of the generation of another. The control unit was formed by two signal generators—the master oscillator and the standard two-channel generator G5-27A, having two outputs with time adjustment for the second signal relative to the first. The first output signal included Nd:YAG laser operating in the free-running mode, and the second signal after the necessary delay turned on the Nd:YAG laser in the Q-switched mode. Changing the amplitude of the output signals of the G5-27A generator allowed the lasers to be switched on singly. The master oscillator provided the operation of the G5-27A generator with a frequency from 1 to 10 Hz. As a result of the combination of beams in the irradiation zone, the complex pulse with an inhomogeneous amplitude–time structure was formed (Figure 2).

As the thermal pulse, Nd:YAG laser radiation was used, operating in the free-running mode (radiation wavelength 1.06 µm, energy E_1_ = 0.2–1.0 J, and pulse duration 500 µs). In the work, the value of 500 µs was experimentally selected as the pulse duration in the free-running mode. The chosen time turned out to be optimal and increased the efficiency of the drilling process of the processed material with a thickness of 1.0 mm, which ensured the release of the maximum amount of condensate. This unconventional radiation pulse duration for laser machining was chosen based on the desire to increase the productivity of the process by increasing the volume of the melt bath (compared with shorter pulses). The value of the delay time of the Q-switched mode relative to the free-running pulse was obtained experimentally. As preliminary experimental studies have shown, in the case when the pulse duration of the free-running mode is less than 500 µs (for example, 350 µs), the delay time for effective removal of the burr from the hole was up to ~340 µs. In the case of increasing the pulse duration to 600 µs, the delay time was ~580 µs. The removal of the melt from the irradiation zone was provided by the powerful vapor pressure pulse, which was formed by the pulse of the Q-switched mode of the second Nd:YAG laser (radiation wavelength 1.06 µm, energy E_2_ 0.1 J, pulse duration 25–30 ns).The laser radiation energy was measured using the specialized LabMax-Top calorimeter with J25MB-HE and PM10 sensors, which was installed at the time of measurement between the partially transmitting mirror (6) and the focusing lens (7). To analyze the structure of the material, the samples were prepared along the plane located along the axis of the hole formed during laser processing. The samples were prepared using laser scribing of ceramic sections adjacent to the holes, followed by mechanical separation of ceramic particles and further grinding. In the manufacture of the metallographic specimen, plane-grinding rotating circles made of polishing material with a highly abrasive diamond paste applied to its surface were used.

**Figure 3:**
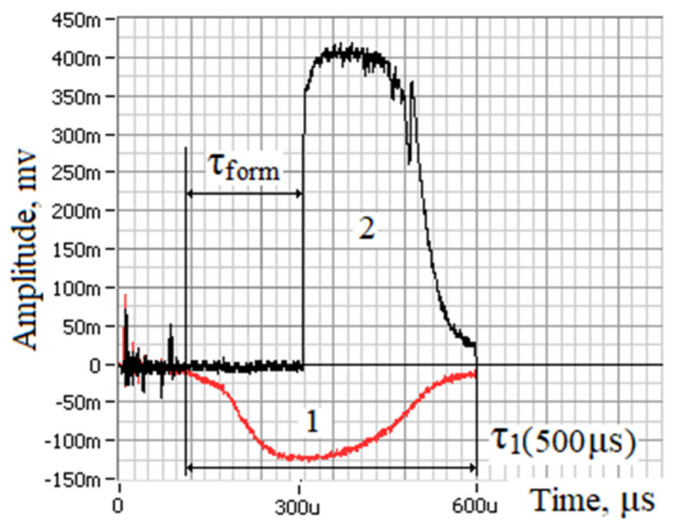
A characteristic view of the oscillogram of laser pulses incident (1) and transmitted (2) through the formed hole.

The experiments were carried out on thin-sheet (0.5–1.0 mm) samples of alumina ceramics in the air at atmospheric pressure. The ceramic samples were fixed on an XYZ coordinate table. The surface of the sample was located perpendicular to the direction of incidence of laser radiation. The laser radiation was focused on the lower surface of the sample into a spot with a diameter of ~0.25 mm. The focal length of the lens is f = 127 mm. A total of 130 holes were formed on each sample at fixed laser radiation parameters and at a certain delay time. The dependence of the mass of the removed material per unit of energy expended on the delay time in the Q-switched mode relative to the beginning of the thermal laser pulse was investigated. After the laser drilling process, the sample was cleaned in an ultrasonic bath with ethyl alcohol for ~30 min, then dried. Dependencies were removed for fixed values of the thermal laser pulse (E_1_ = 0.2; 0.3; 0.4; 0.5; 0.8; 1.0 J); the amount of material to be removed was determined by measuring the mass loss of samples after repeated (100 times) exposure to the target on Sartorius Cubis II analytical scales with an accuracy of 0.001 g. The study was carried out for several values of the energy of a thermal laser pulse. The minimum free-running pulse energy required for drilling and the duration of the hole formation process in samples with different thicknesses were determined experimentally, for which a fairly simple technique was proposed. As shown in Figure 1, the diagram shows two photodetectors connected to the digital oscilloscope TDS 2004B. The selective filters were installed in front of the photodetectors, passing only the wavelength of the laser radiation λ = 1.06 microns. The oscilloscope worked in the standby mode and was started using an incident pulse of radiation. The first one (1) is located on the Nd:YAG laser side (worked in the free-running mode) and registers an incident pulse of laser radiation. The second (2) photodetector is located behind the back surface of the flat sample, on the same axis with the radiation focusing spot. At the moment of the hole formation (at a certain radiation energy), the photodetector (2) registers the radiation pulse that has passed through the formed hole. This makes it possible to determine the value of the radiation energy required for the formation of the hole and the threshold penetration time to the full depth (the time for which the hole is formed) τ_form_ (Figure 3). 

## 3. Results and Discussion

**Figure 4:**
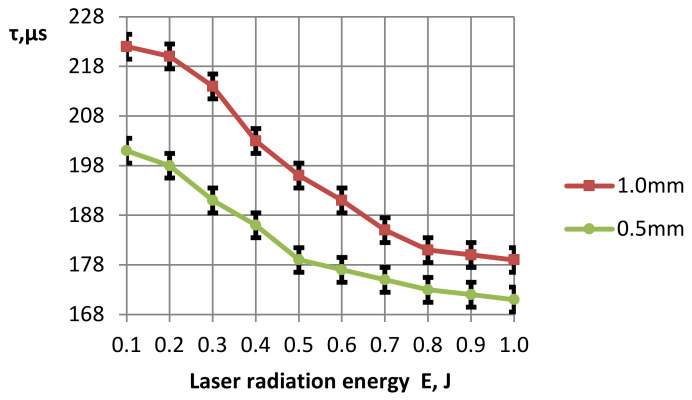
The dependence of the drilling time in sheet ceramics at different thicknesses on the laser radiation energy (τ_1_ ≈ 500 µs, the area of the focus spot S_p_ ≈ 0.25 mm^2^).

According to the considered method, experiments were carried out to determine the time of hole formation using millisecond laser radiation in aluminum ceramics with the thickness of 0.5 and 1.0 mm at different values of radiation energy and a fixed value of the area of the laser radiation focusing spot at the constant pulse duration in the free-running mode. The results of the conducted studies are presented in Figure 4.

Figure 4 shows that for small values of the thermal pulse energy (0.2; 0.3; 0.4; 0.5 J), the nature of the change in the drilling time in ceramic plates with the thickness of 0.5 and 1.0 mm is the same—the difference between the time remains unchanged (≈20 μs). With the increase in energy to 0.8; 1.0 J, the difference between the drilling time on plates of different thicknesses (0.5 and 1.0 mm) also decreases (≈10 μs). At the same time, the hole formation time is reduced by 19% for the thickness of 1.0 mm and by 14% for a thickness of 0.5 mm. This is due to the intensification of melting and evaporation processes in the forming channels.

**Figure 5:**
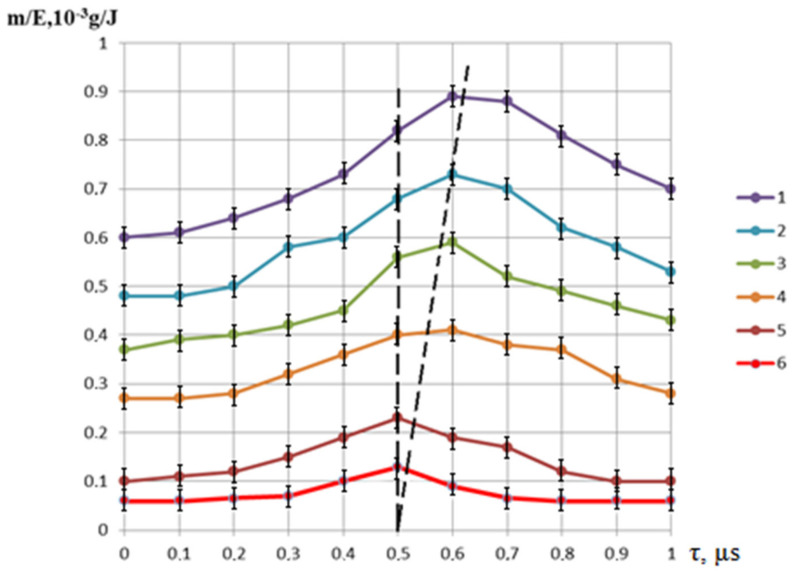
The dependence of the mass of the material to be removed per unit of energy expended on the pulse delay time in the Q-switched mode relative to the onset of the thermal laser pulse E_1_: 1—1 J; 2—0.8 J; 3—0.5 J; 4—0.4 J; 5—0.3 J; 6—0.2 J.

**Figure 6:**
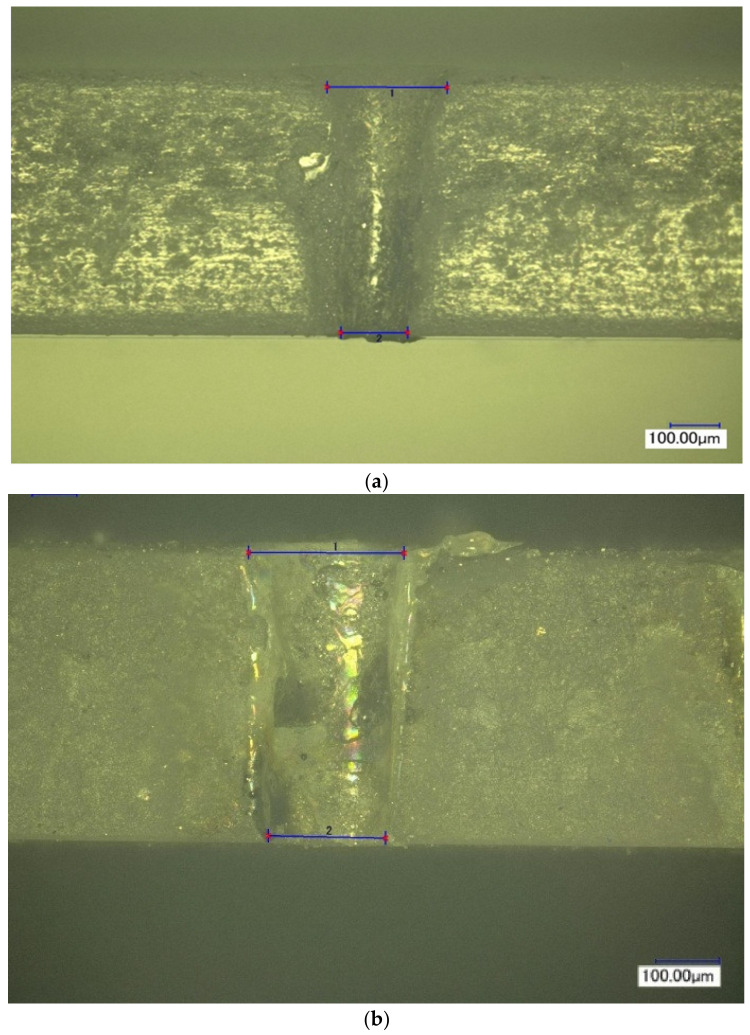
The appearance of the hole formed with laser radiation operating in the modes: free-running mode: τ_1_ = 500 μs, E_1_ = 0.2 J (**a**); two-stage mode: τ_1_ = 500 μs, E_1_ = 0.2 J, τ_2_ ≈ 25–30 ns, E_2_ = 0.03 J (**b**).

**Figure 7:**
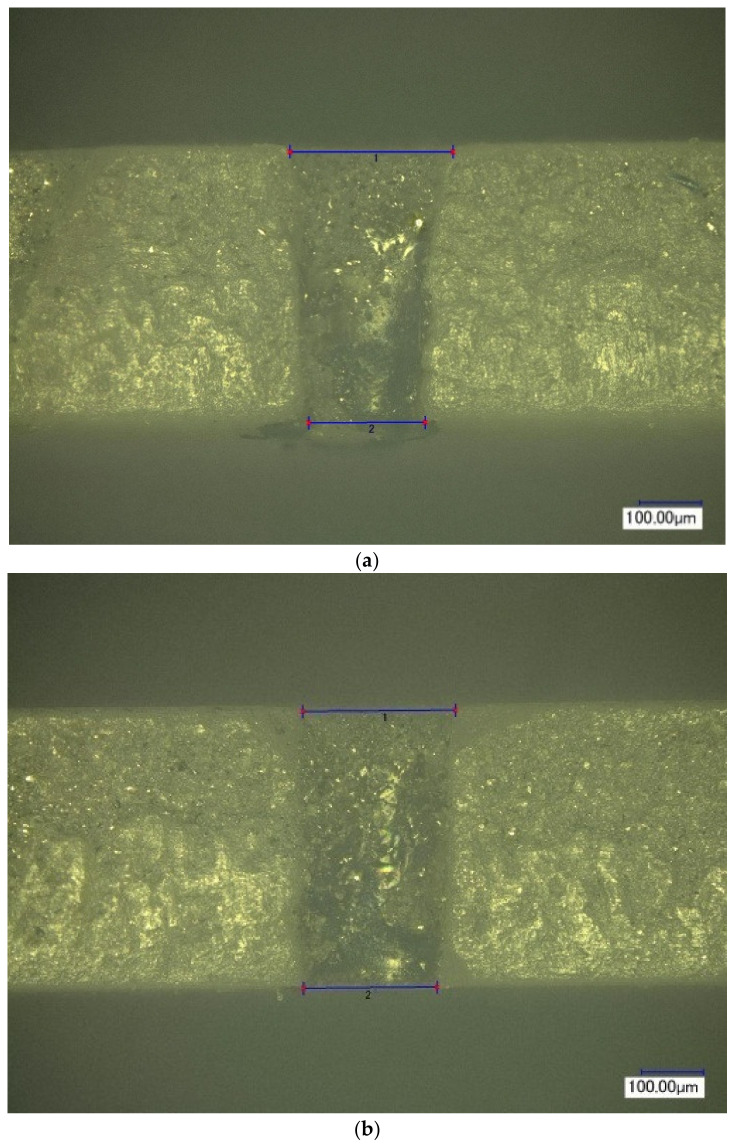
The appearance of the hole formed with laser radiation operating in the modes: free-running mode: τ_1_ = 500 μs, E_1_ = 1.0 J (**a**); two-stage mode: τ_1_ = 500 μs, E_1_ = 1.0 J, τ_2_ ≈ 25–30 ns, E_2_ = 0.03 J (**b**).

**Figure 8:**
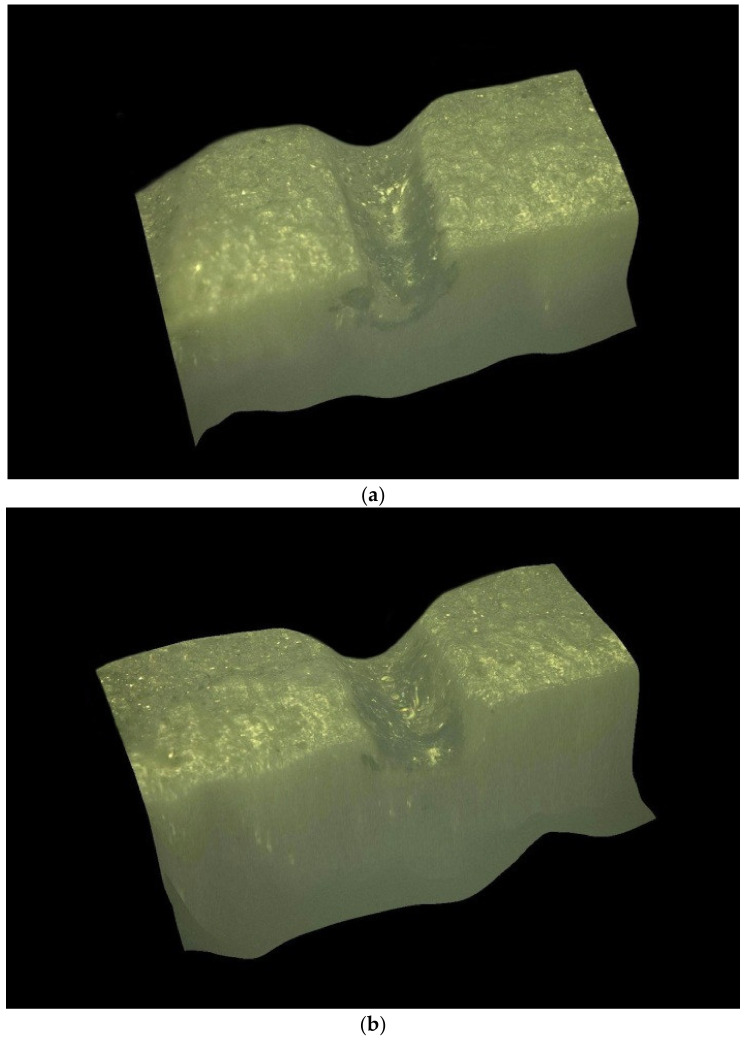
A 3D image of a hole formed with laser radiation operating in the following modes: free-running mode: τ_1_ = 500 μs, E_1_ = 1.0 J (**a**); two-stage mode: τ_1_ = 500 μs, E_1_ = 1.0 J, τ_2_ ≈ 25–30 ns, E_2_ = 0.03 J (**b**).

**Figure 9:**
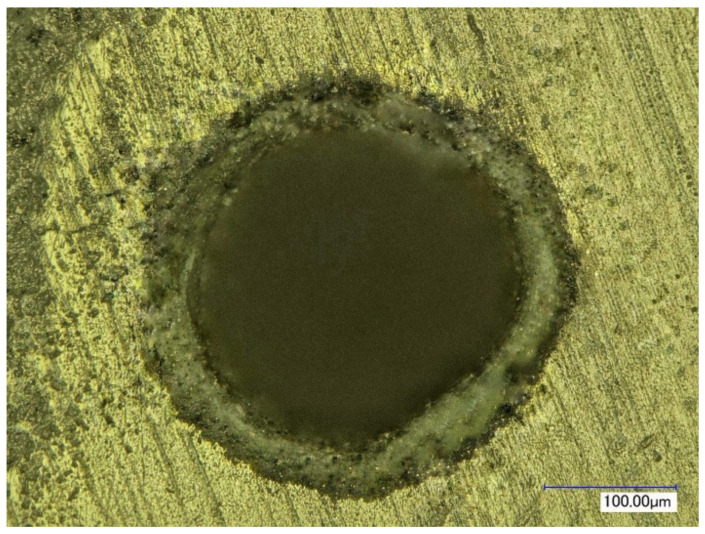
The top view of the hole formed with laser radiation operating in the two-stage mode.

Figure 5 shows the normalized per unit energy dependences of the mass of the removed material on the pulse delay time in the Q-switched mode of the Nd:YAG laser relative to the onset of the thermal pulse in the free-running mode of the Nd:YAG laser. It was noted that for relatively small pulse energies of thermal action (E_1_ up to 0.3 J), the maximum position occurs at the end of the thermal pulse. With increasing energy, there is a certain displacement of the maximum in time, due to the fact that the melting zone of the material continues to progress even after the action of the thermal pulse, due to the fact that the average value of the melt temperature by the end of irradiation significantly exceeds the melting temperature of the material. With the increase in the energy of the thermal laser pulse, the effect of radiation in the Q-switched mode on the process of removing material from the melt bath decreases markedly. This is clearly noticeable by the change in the ratio of the mass of the removed condensate to the value of the same mass only under thermal laser exposure (free-running mode). The decrease in the influence of the pulse in the Q-switched mode is manifested in the fact that, with an increase in the energy of the main pulse, partial evaporation of the melt becomes possible directly due to the thermal pulse, and only the removal from the walls of the formed hole of the remaining part of the melt remains for the share of the additional pulse. In addition, this is caused by the shielding of radiation with the vapor plasma torch, which occurs at sufficiently high-pulse intensities of thermal action. The appearance of the hole was studied using a KEYENCE VHX-1000 optical microscope and is shown in Figure 6, Figure 7, Figure 8 and Figure 9. Figure 8 shows the formed holes in the 3D image obtained on the optical microscope.

In the resulting hole, formed when exposed to laser radiation in the free-running mode, individual condensate particles of the processed ceramics of a flake shape were found. Their occurrence is caused by the process of melting and the separation of melted ceramic particles under the action of the pulse of laser radiation in the free-running mode, Figure 6a and Figure 7a.

When using laser treatment in the two-pulse mode, the emission of dispersed ceramic particle residues in the formed channel was much less than with millisecond exposure, as shown in Figure 6b and Figure 7b.

The average diameter of the inlet and outlet holes after exposure to laser radiation in the free-running and Q-switched mode was 253 μm and 209 μm, respectively.

The results of processing the geometric parameters from the obtained holes showed that the taper after drilling in the free-running mode is more than that after two-stage exposure.

In a previous research work [26], the procedure has been experimentally and theoretically demonstrated that makes it possible to significantly improve the degree of taper of laser drilling by using a nanosecond two-pulse laser beam with a double wavelength. At the same time, the values of the taper holes obtained by the authors of a previous study [26] are significantly lower compared to similar parameters obtained in this study.

In another study [28], when a millisecond Nd:YAG laser is applied to alumina ceramics with a longer duration of the radiation pulse, a wider HAZ is formed in the hole than that obtained in this work, which can lead to the formation of microcracks on the side surface due to a large temperature gradient.

The results of the study [20] showed that drilling in thin-sheet ceramics with Nd:YAG laser radiation in the free-running mode with the parameters—pulse duration 0.5 ms, energy 1.47 J, frequency 20 Hz—leads to the formation of holes with a good taper, which is comparable with the results of this work, but with high energy consumption.

In addition, studies were carried out on the processing of the same materials according to the scheme proposed previously [36], which implemented the scheme for switching the single laser pulse of radiation from the free-running mode (thermal pulse) to the Q-switched mode, consistent with the process of the beginning of the hole formation. The study of the laser machining of alumina ceramics according to this scheme showed similar results with the two-pulse scheme on samples up to 1 mm thick. With large thicknesses of Al_2_O_3_ sheet ceramics, the laser machining characteristics are more effective when using the two-pulse circuit shown in Figure 1.

The comparison of the processing results, expressed in the noticeable decrease in the amount of burr and influx inside the cut spaces, confirms that the considered laser machining mode, both quantitatively and qualitatively, is noticeably superior to the traditional mode drilling in ceramics. This determines the prospects of using the proposed method in the technologies of laser machining of ceramic materials in the production of commodities in the electronic and radio industries.

## 4. Conclusions

1. The combination of pulses in the free-running and Q-switched modes makes it possible to significantly increase the efficiency of laser machining of ceramics.
2. The method of determining the minimum energy required for the formation of holes in thin-sheet ceramics and the time of hole formation has been implemented in practice.
3. The values of the minimum free-running pulse energy necessary for the formation of a hole (with a ceramic thickness of 1 mm) were experimentally determined as follows: E_1_ = 0.2 J and the hole formation time (τ = 220 µs).
4. The optimal value of the pulse delay time in the Q-switched mode relative to the pulse in the free-running mode, providing high efficiency with the maximum amount of the removed condensate and the thickness of the processed material 1.0 mm, was equal to the pulse duration in the free-running mode of 500 µs.
5. As a result of the conducted experimental studies, the parameters necessary for the more detailed description of the processes of laser machining of aluminum ceramics with the complex amplitude–time structure have been obtained, and they can be used both in theoretical models and for the development of technologies for laser machining of ceramic materials.
